# Diagnosis and Managment of Maxillary Incisor with Vertical Root Fracture: A Clinical Report with Three-Year Follow-Up

**DOI:** 10.1155/2018/4056390

**Published:** 2018-02-06

**Authors:** Ines Kallel, Eya Moussaoui, Fadwa Chtioui, Nabiha Douki

**Affiliations:** ^1^Department of Dental Medicine, Faculty of Dentistry, Hospital Sahloul, Sousse, Tunisia; ^2^Laboratory of Research in Oral Healh and Maxillo Facial Rehabilitation (LR12ES11), Monastir, Tunisia; ^3^Faculty of Dental Medicine, University of Monastir, Monastir, Tunisia

## Abstract

According to the American Association of Endodontists, “a ‘true' vertical root fracture is defined as a complete or incomplete fracture initiated from the root at any level, usually directed buccolingually.” Vertical root fracture (VRF) usually starts from an internal dentinal crack and develops over time, due to masticatory forces and occlusal loads. When they occur in teeth, those types of fractures can present difficulties in diagnosis, and there are however many clinic and radiographical signs which can guide clinicians to the existence of the fracture. Prognosis, most often, is hopeless, and differential diagnosis from other etiologies may be difficult sometimes. In this paper, we present a case of VRF diagnosed after surgical exploration; the enlarged fracture line was filled with a fluid resin. A 36-month clinical and radiological follow-up showed an asymptomatic tooth, reduction of the periodontal probing depth from 7 mm prior to treatment to 4 mm with no signs of ankylosis. In this work, the diagnosis and treatment alternatives of vertical root fracture were discussed through the presented clinical case.

## 1. Introduction

A vertical root fracture (VRF) is a root fracture extending along the longitudinal axis of the root; it can be divided into complete and incomplete vertical root fractures based on the separation of the two fragments according to Leubke's classification [1], and it is often observed in endodontically treated teeth. Communly, VRF initiates from the internal root canal wall and extends to the outer root surface, usually in a buccolingual direction. The fracture might involve one surface (buccal or lingual) or both surfaces (buccal and lingual).

These types of fractures can affect either the root or extend coronally towards the cervical periodontal attachment [2, 3]. According to some studies [2–4], they are the third most common cause of tooth loss after dental caries and periodontal disease. VRFs have been reported to occur in both nonendodontically and endodontically treated teeth. The latter represents but the majority of the cases, with or without post insertion.

A retrospective study showed that 94% of teeth with root fractures have a history of endodontic treatment [4]. The prevalence of VRF in endodontically treated teeth (ETT) ranges from 2% to 20% [5]. From a biomechanical standpoint, this condition has been associated with a synergism between chemical and mechanical degradation of the tooth [6].

Patients with VRFs typically present with minimal signs and symptoms during the early stage. Consequently, the entity is generally not noticed until periapical pathology occurs. Under such circumstances, the diagnosis is difficult, as they mimic other conditions [3]. In up to 67% of all root-filled teeth with VRF, symptoms or signs include localized swelling, pain on biting, associated with a fistula or sinus tract, sensitivity to percussion and palpation, and deep localized periodontal probing pocket depth.

Radiographic observation of an angular pattern of apical or lateral bone resorption with a radiolucent halo is considered pathognomonic [7].

Many clinical studies have investigated the different diagnostic and clinical parameters associated with VRF. The use of cone beam computed tomography (CBCT) to identify the presence of VRFs [8, 9] was recommanded in many studies. *Their diagnostic accuracy, nevertheless, is still uncertain* (but there is still a considerable uncertainty regarding their diagnosis) [9, 10].

The prognosis of such teeth is generally questionable and the extraction of the tooth being the most common treatment option. However, conservative treatment options such as reconstruction of the fractured fragments with adhesive resin followed by intentional replantation have been recently suggested [11].

The present case report describes the difficulty of diagnosis of an incomplete vertical root fracture in a maxillary right central incisor with a successful management by sealing the fragments with a fluid resin composite. At the three-year follow-up, the tooth was asymptomatic, radiographically sound with probing depth and mobility within normal physiological limits.

## 2. Case Report Presentation

A 14-year-old male was referred to the Department of Dental Medicine complaining of occasional pain and a mucous fistula which persisted for one year in his upper right central incisor. When history was elicited, the patient revealed that there was a fall two years back in school due to which he sustained fracture of his maxillary permanent central incisors. Both teeth underwent endodontic treatment in a private office. According to his medical history, the patient exhibited no systemic disease.

**Figure 1:**
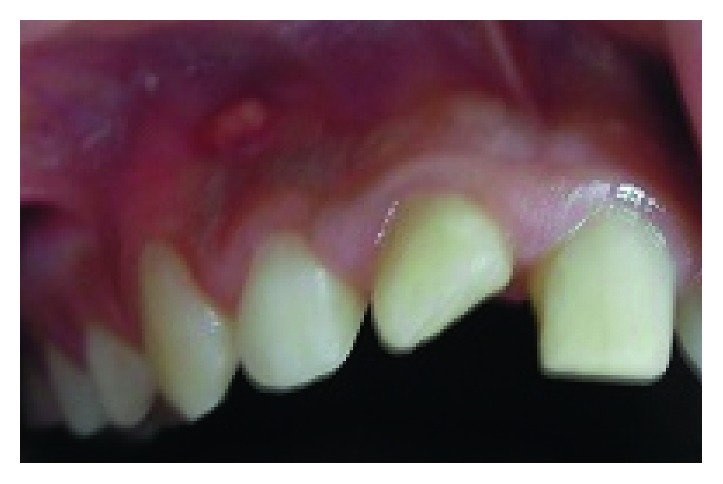
Endobuccal examination: mucosa fistula.

**Figure 2:**
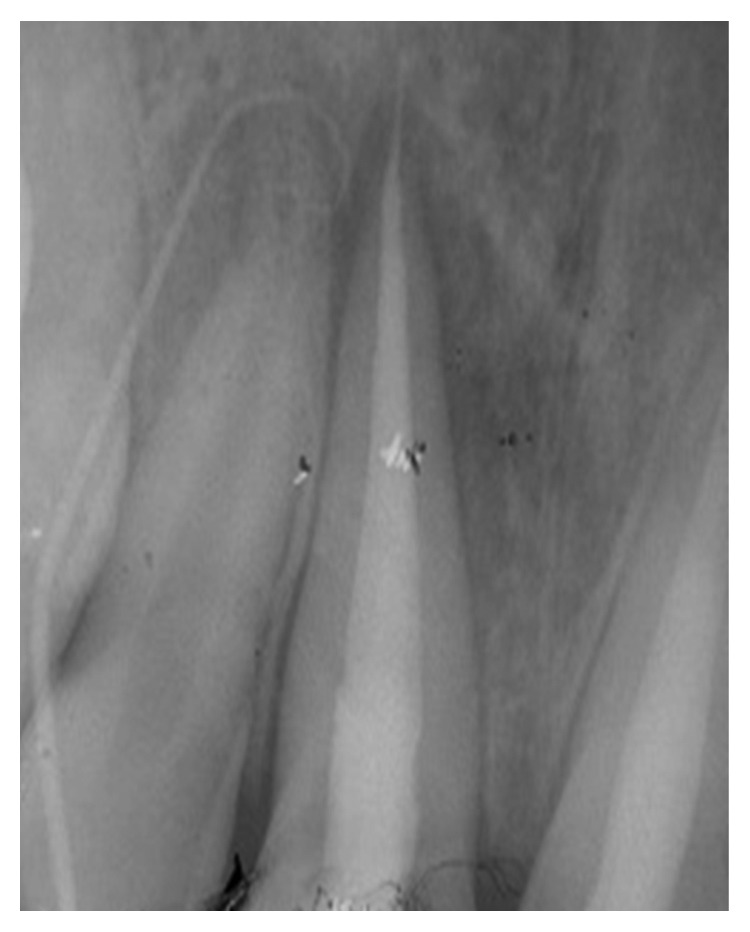
Radio using locating cone showing the relationship with tooth 12. Teeth 11 and 21 were both endodontically treated.

**Figure 3:**
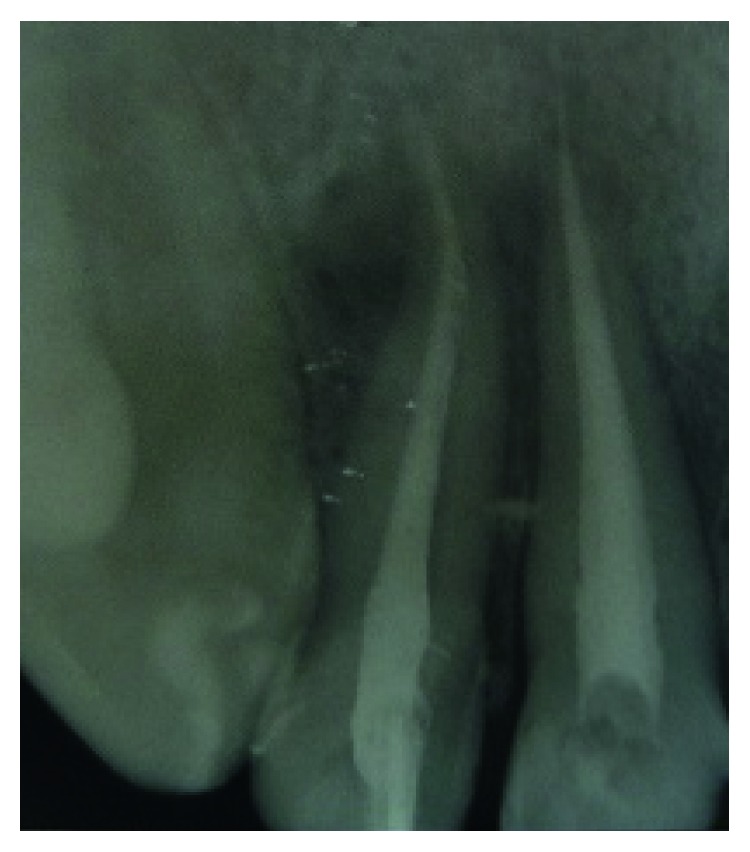
Root canal filling of tooth 12.

**Figure 4:**
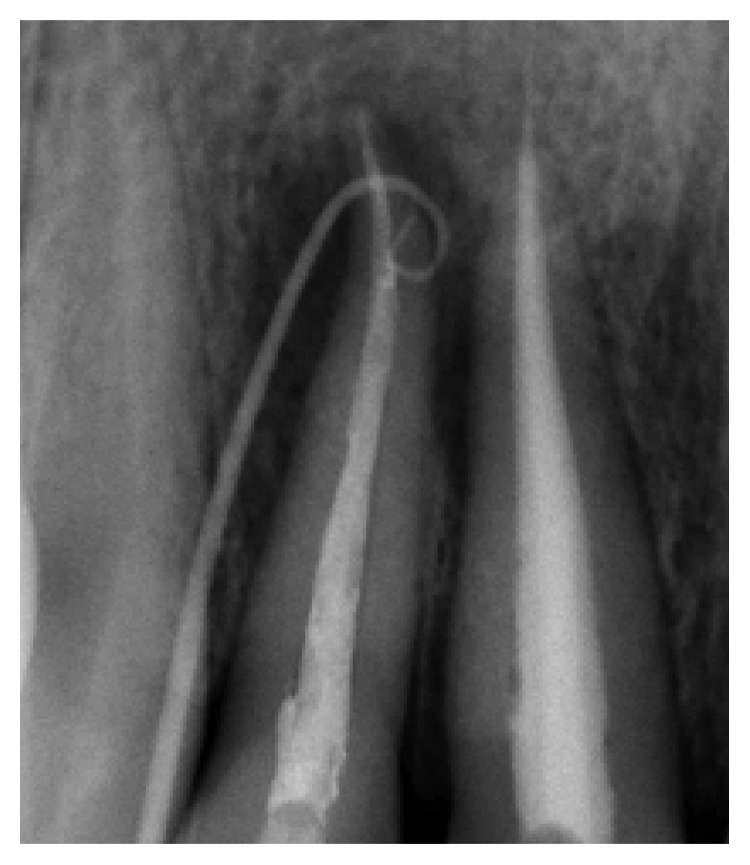
Fistula recurrence after 2 years involving tooth 12, persistence of periapical lesion.

**Figure 5:**
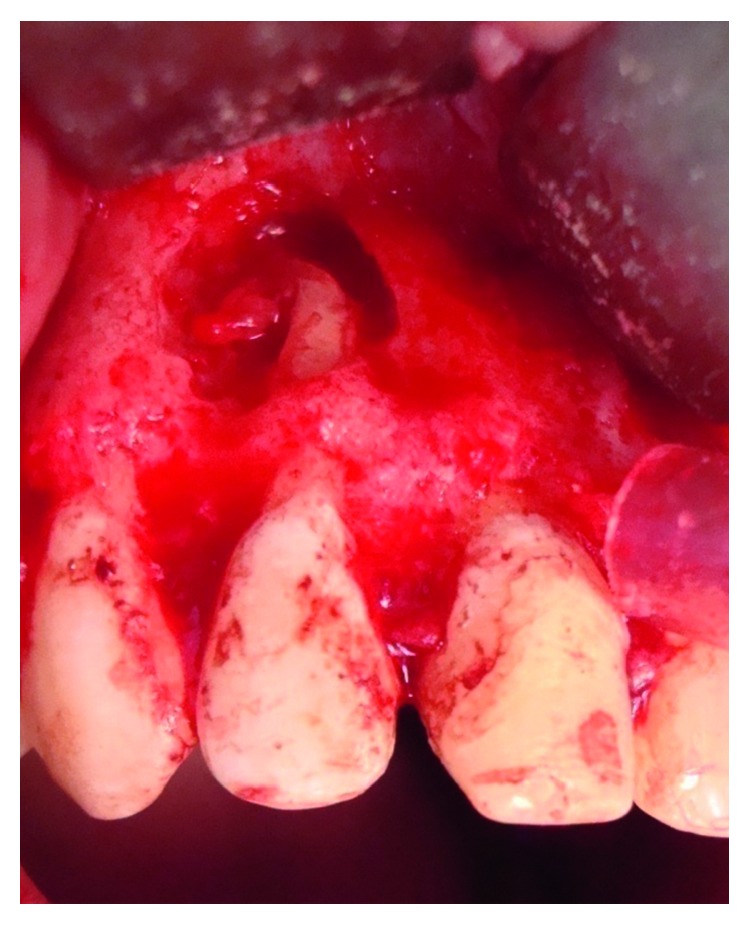
Presence of granulation tissue on the periapical side of tooth 12.

**Figure 6:**
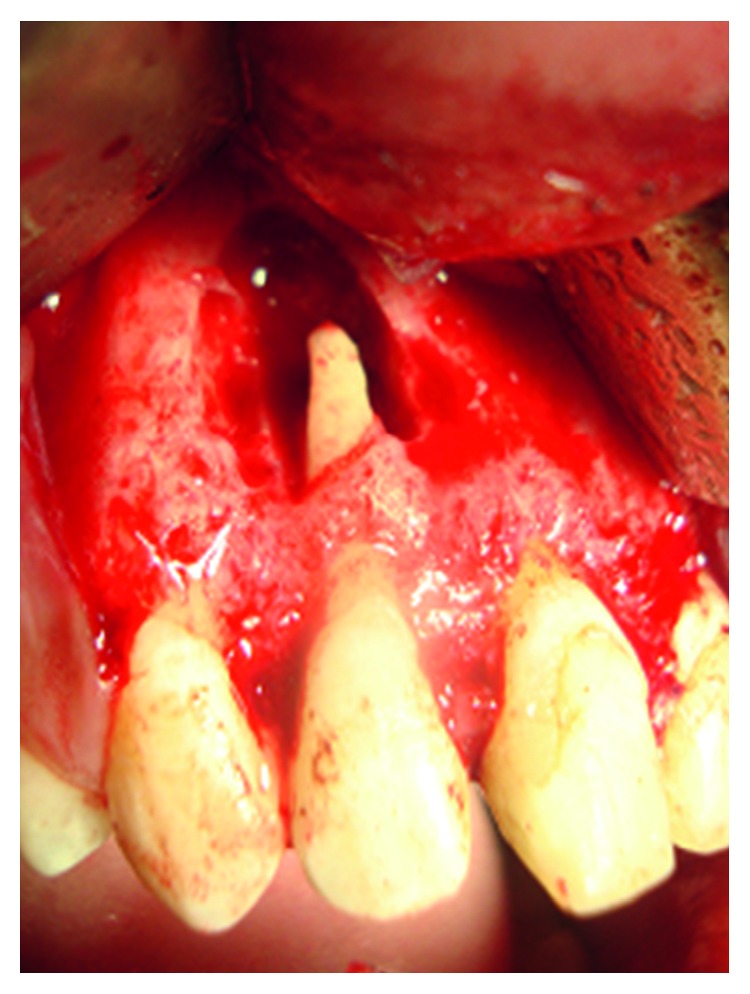
Apical root resection.

**Figure 7:**
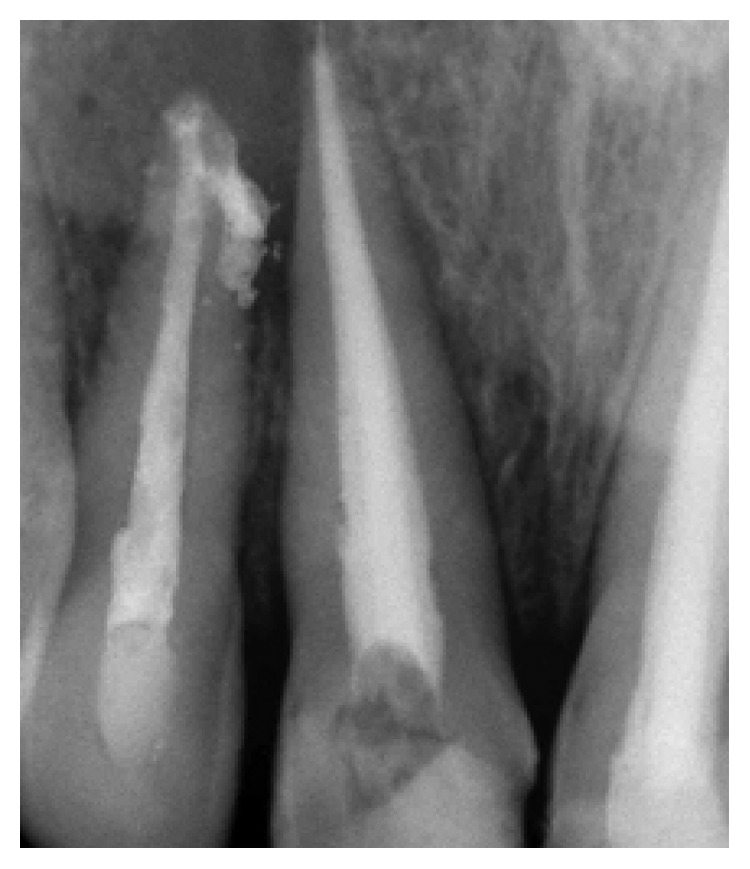
Radiological control after root end filling: excess glass ionomer cement.

His extraoral examination was noncontributory. Intraoral examination revealed enamel-dentin fracture involving the incisal edge in tooth #11, in the upper right lateral incisor region (Figure 1). Periodontal probing revealed deep narrow pockets on both facial of tooth 12 and palatal root surfaces of tooth 11 of 5 and 6 millimeters, respectively, while the other teeth showed a nonpathologic probing depth of value of 2-3 mm. Pulp sensibility tests showed a negative response in tooth #12. Radiographic evaluation was performed using a film holder which showed the relationship with tooth #12; both central incisors were endodontically treated (Figure 2). Endodontic treatment of tooth #12 was planned (Figure 3). A 3-month follow-up appointment and coronal restoration of teeth #12 and 11 was scheduled, followed by another appointment 3 months later, but the patient consulted us only 2 years later complaining of the recurrence of the Fistula. A periapical radiograph with film holder has built evidence to show the relation with tooth #12 (Figure 4). A Periapical surgery was carried out and revealed a proliferation of granulation tissue around the apex of the lateral incisor (Figure 5). Periapical curettage followed by a 3 mm root-end resection of tooth #12 (Figure 6) was conducted. After Retrograde root preparation, retrograde filling was performed with glass ionomer cement. The radiological control after root-end filling showed cement excess (Figure 7).

**Figure 8:**
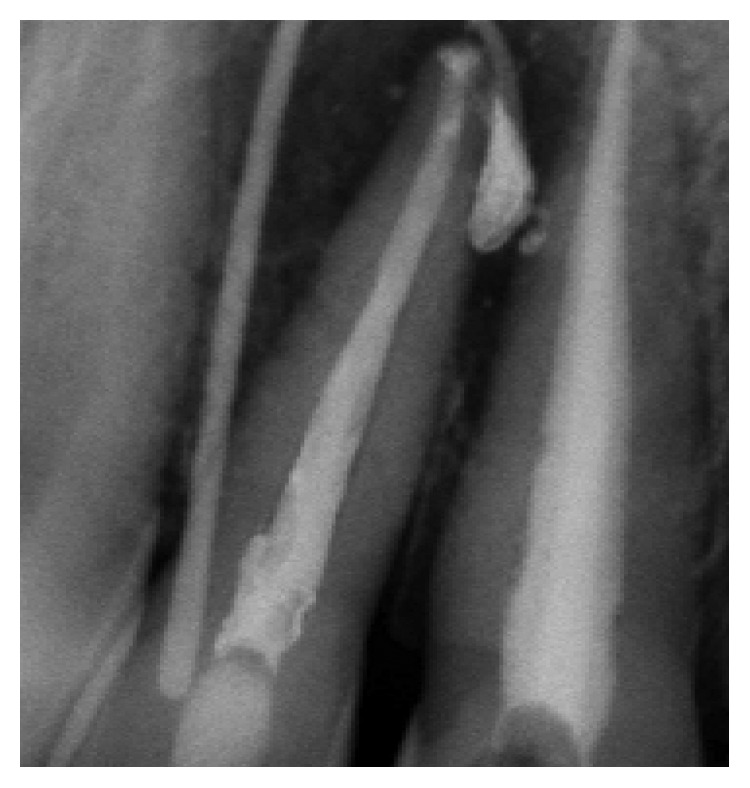
Fistula recurrence after 3 weeks. Locating cone showed relation between tooth 12 and tooth 11.

**Figure 9:**
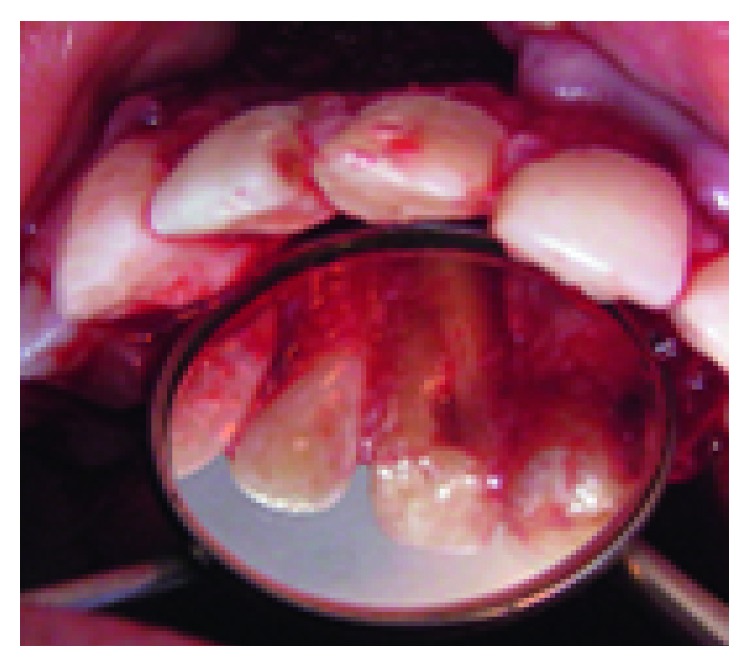
Second surgical exploration showing vertical root fracture in the palatal side of tooth 11.

A follow-up appointment was given 3 weeks later. The reappearance of the sinus tract was actually surprising; a periapical radiograph using gutta-percha cones, after fitting them to a gauged apex, showed a relation between both right lateral and central incisors (Figure 8). A second exploratory surgery at routine six-month recall was decided with palatal and buccal full-thickness flaps elevation, revealing the undiagnosed VRF on the palatal side of tooth #11(Figure 9).

**Figure 10:**
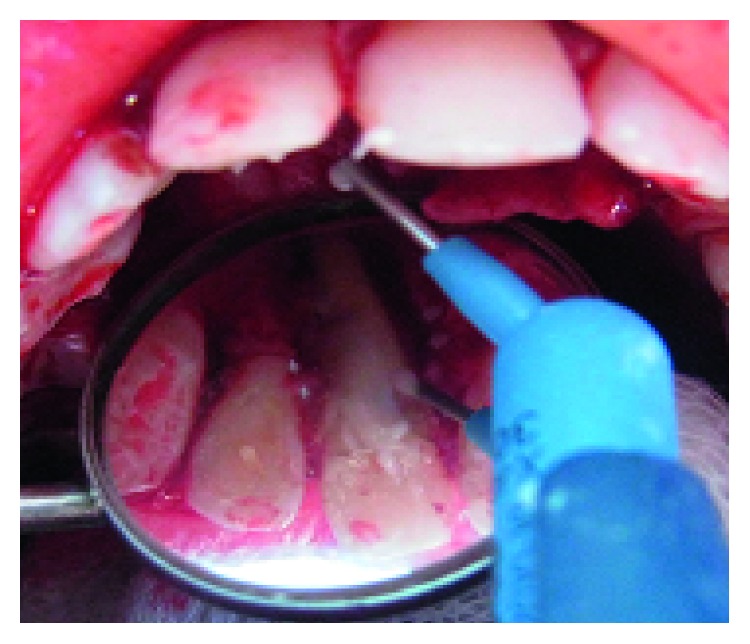
Sealing of the vertical root fracture with fluid resin composite.

We also noted an extended bone loss from the vestibular to palatal side. The alveolar socket was carefully curetted to remove all granulation tissue. The fracture line was sealed using a fluid composite resin (Figure 10).

**Figure 11:**
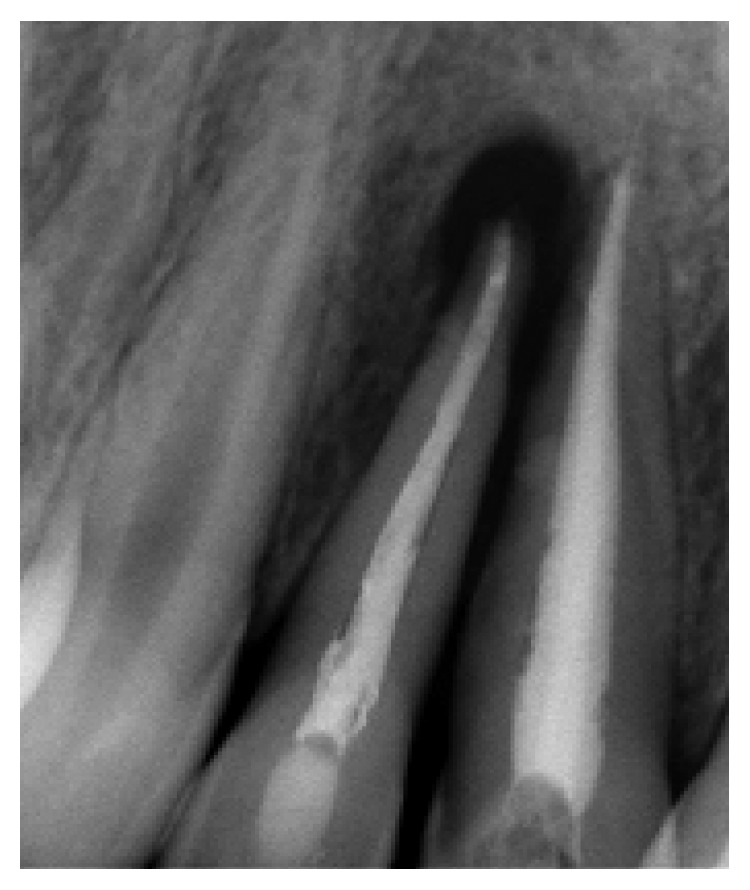
Radiograph of control after 1 month.

**Figure 12:**
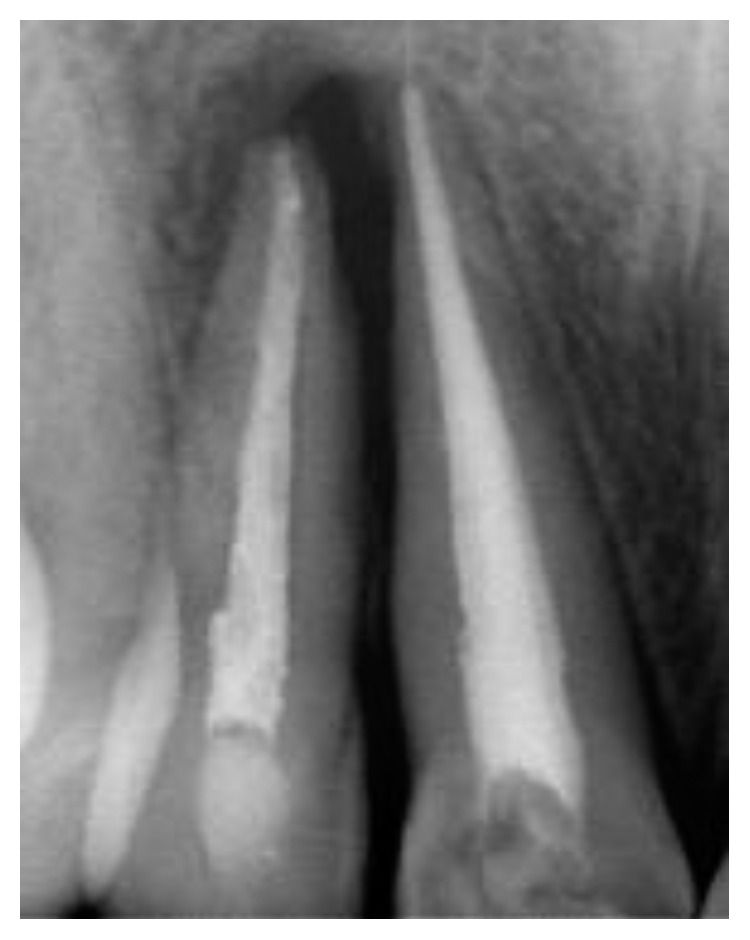
Radiograph of control after 3 months.

**Figure 13:**
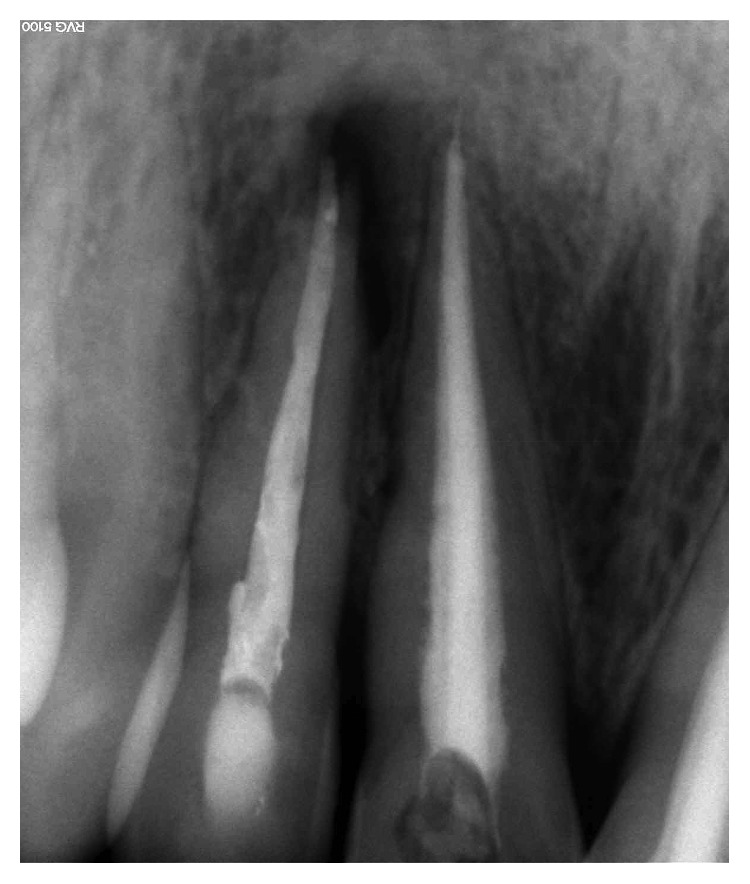
Radiograph of control 1 year later.

Clinical and radiographic evaluation was performed after one month (Figure 11), then three months later (Figure 12), and at one-year follow-up (Figure 13). Regular six-month follow-ups were scheduled afterwards.

**Figure 14:**
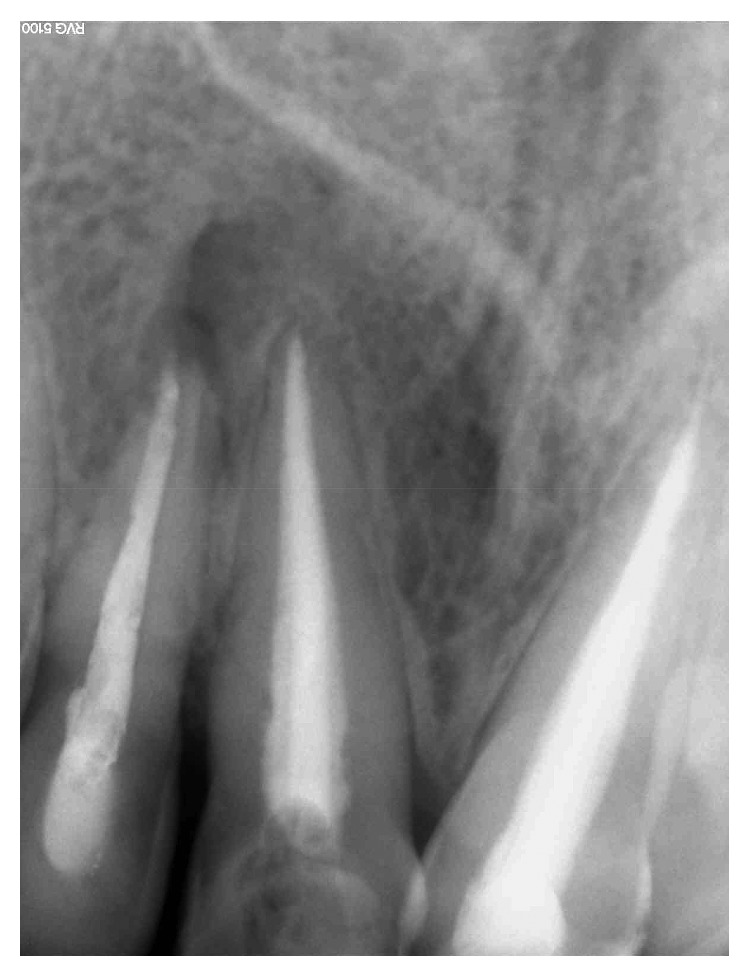
Radiograph of control 3 years later.

After three years (Figure 14), the healing of tooth #11 was evident with a probing depth anda mobility ranging within normal physiological limits. No radiographic signs of resorption were observed.

## 3. Discussion

The retention of microbial dental plaque in these difficult-to-clean areas has been shown to be associated with local periodontal inflammation and periodontal destruction, which is one of the reasons why deep probing pocket against the fracture line is the most common feature of VRF; however, it remains surrounded by normal pocket depths. This may also involve the appearance and recurrence of the fistula, typically located close to the gingival margin, as it was reported in our patient's case. Those characteristics appear because there is bone resorption surrounding the fracture line on the bone plate [12].

Lustig et al. [13] showed after studying 110 vertical fracture cases that the resorption is a consequence of a chronic inflammatory process resulting in a granulation tissue that comes replacing the bone following a bacterial infection. The latter subsequently gains an easy passage through the fracture line bypassing the defense line of the epithelial attachment.

Although one may argue about the viability of the described procedure, and the degradation of the periodontium and osseous architecture during the recall period if the procedure was not successful, periodontal pockets exceeding 6 mm do not necessarily indicate imminent deterioration, and the sites with such deep probing depths may be maintained successfully for a long period by personal plaque control and professional cleaning. This may explain the successful outcome of the vertically fractured tooth in our case treated by flow resin (ceram x duo DENTSPLY) with residual pockets [14, 15].

This case report presents success even after a period of three years of follow-up as the tooth showed no clinical symptoms, demonstrating periodontal tissue healing/regeneration, and improvements in periodontal probing at the fracture site.

### 3.1. Difficulty in Diagnosis

Vertical root fractures are most commonly associated with endodontically treated teeth; their presence in a nontreated tooth is rare. There is no single clinical feature that indicates their presence. They are also difficult to diagnose, as they mimic other conditions. Hence, the diagnosis of vertical root fractures requires more of a predictive rather than a definitive identification. A cumulative assessment of the clinical signs and symptoms and the radiographic features may help us reach a definitive diagnosis [11].

Some clinical tests may guide us towards the right diagnosis of VRF. For instance, periodontal probing may reveal a narrow, isolated, periodontal defect within the gingival attachment.

Tracing the sinus tract using gutta-percha as an endodontic explorer may be used to trace the sinus tract back to its origin, but in our clinical case, we were confused with the periapical lesion of the adjacent tooth.

Bite test, such as biting cotton wool rolls or wood sticks, may be used to reproduce the pain on biting described by the patient. This test is performed tooth-by-tooth or cusp-by-cusp in multirooted teeth. Usually, the patient feels relaxed on biting, and the pain starts while releasing the biting pressure.

Radiographic examination associated with clinical features can lead to a positive diagnosis, and many radiographic features are possible:

- Radiolucent lines along the root fillings or posts: appearance of a vertical space adjacent to the root filling material or a space between the edges of a root canal.
- Direct evidence of the fracture line is often difficult to visualize. To be able to see the fracture, the X-ray beam must pass almost directly down the fracture line.
- Fracture line deviating from the long axis of the canal may be radiographically more obvious, as compared to the fracture line running parallel and adjacent to a root filling.
- Double images: when separation of the fragments occurs in a direction other than parallel to the X-ray beam, overlapping of the fragments may result in double images of the external root surface. However, this effect is sometimes seen in normal teeth, for example, in the mesial concavity of maxillary premolar teeth.
- Extrusion of cement or filling: extrusion of cement or root filling material may occur into the fracture site or apically when the fracture is present prior to filling. It may also occur during root filling procedure.
- Widening of the periodontal ligament space: along the whole length of the root may indicate VRF. It is different from bone loss seen in a periapical lesion where it occurs apically but without destruction of the lamina dura along the root surface.
- Radiolucent halos: “halo-like” radiolucency running along the whole length of the root surface is a classic sign of VRF.
- Step-like bone defects: oblique fractures often lead to a characteristic step-like bone defect which may mimic endodontic lesions.
- Isolated horizontal bone loss in posterior teeth.
- Unexplained bifurcation bone loss: furcation bone loss may occur in molars with root fracture, in the absence of apical pathosis or over a periodontal disease and without any apparent reason like root perforation.

The radiologic signs are highly nonspecific and not detectable during the early stages, in which there are subtle fissures with no separation and those develop late as sequelae of chronic inflammation induced by the fracture. Approximately only one third of the fractures may be visualized directly on conventional dental radiographs. Mesiodistally oriented fractures are not visualized directly in a typical radiographic examination.

Current research has employed cone-beam computed tomography (CBCT) to identify the presence of VRF [8, 9]. Unfortunately, root fractures are difficult to assess, because most of them occur in teeth with RCT. Therefore, the superposition of the fracture line with the filling material decreases the diagnostic accuracy [10].

Currently, there is no evidence supporting the use of CBCT to detect VRF in ETT. Therefore, the diagnosis should be confirmed after surgical flap elevation [3].

An exploratory surgical procedure helps in the definitive diagnosis, if VRF is strongly suspected from the clinical and radiographic signs. Gentle soft tissue retraction may be sufficient to view the fracture line, and a dye material may also be used for a better visualization of the fracture line. As a probe is passed over the fracture line, “clicking” sound may be heard. Reflection of a small full-thickness flap may be required in some cases. This was the case for our patient where diagnosis was made only after a surgical exploration of the side of the vertical fracture, especially that the real cause was hidden by the presence of another periapical lesion of the adjacent tooth which misled us from the correct diagnosis.

### 3.2. Etiologies

The etiology of VRFs is multifactorial and can be divided into predisposing and iatrogenic factors.

#### 3.2.1. Predisposing Factors for Endodontically Treated Teeth

- Root anatomy: roots with narrow mesiodistal diameter, root curvatures [17, 18], and depressions in the mesial root of mandibular molars as well as in buccal roots of bifurcated maxillary premolars predispose them to fracture, especially at a later stage when additional tooth structure is removed during root canal and dowel space preparations.
- Moisture loss in pulpless teeth [1] was reported to make endodontically treated teeth more brittle. However, this finding was not supported in some studies [19, 20].
- Loss of bone support due to periodontal disease and preendodontic and prosthetic treatment can result in reduced ability of the tooth to withstand functional stresses.
- Preexisting cracks.
- Biochemical properties of root dentin: a study on stress-strain response in human dentin showed that the dentin adaptation to functional stress-strain distribution results in a greater mineralization in the buccolingual areas. This may increase the likelihood for a fracture to propagate in this direction, compared with less mineralization and more collagen in the mesiodistal areas [16, 21].

#### 3.2.2. The Iatrogenic Cause of VRF Is Mainly Attributed to Different Phases of Root Canal Treatment

- Loss of healthy tooth substance: combined with tooth loss due to caries, the result of intraradicular procedures, the remaining tooth structure is directly related to the ability of endodontically treated tooth to resist fracture [18, 22].
- Change in the architecture of an endodontically treated tooth makes the tooth more prone to fracture and thus requiring a restoration (full cuspal coverage) that will protect the tooth during function.
- Excessive cutting during various phases of root canal treatment.
- Increased stress generated from threaded and tapered posts.
- Increased wedging forces with lateral compaction of gutta-percha accounts for 48% to 84% of VRFs. The development of these stresses stands behind crack initiation and propagation, leading eventually to root fracture [17, 23].

Fracture occurring directly during root canal treatment as a result of excessive force application is rare as the required forces for root fractures vary according to the tooth type (approximately 10 to 12 kg) and are well above those that are clinically relevant (1 to 3 kg) during root canal treatment. However, stress caused in dentin may initiate dentinal cracks, which can extend to complete fractures under a functional load. Thus, the multifactorial nature of VRFs has inspired studies in two directions.

Primarily, theoretical research has evaluated the effects of mechanical wear on the dentin in ETT with curved roots and oval channels. Ex vivo studies have associated the presence of isthmus and irregularities in the root canal posterior to mechanical instrumentation with the occurrence of VRF. Whereas in vitro studies have assessed the effects of irrigating solutions, the loss of dentinal moisture after RCT, and the role of tooth restoration and its capacity to respond to masticatory stress on the presence of VRF in ETT.

Secondly, clinical research has investigated factors, such as the type of endodontic treatment and the presence of posts.

A recent study determined that teeth exhibiting dense overfilled root canals significantly increased the odds for VRF by 2.72 times. Another in vitro study of Devale [18] evaluated the effect of instrumentation length and instrumentation systems, and Hand versus Rotary Files on Apical Crack Formation concluded that there was no statistical significance between stainless steel hand and rotary files in terms of crack formation.

Instrumentation length had a significant effect on the formation of cracks when rotary files were used. Using rotary instruments at 1 mm short of the apical foramen caused less crack formation. There was, however, no statistically significant difference in the number of cracks formed with hand files at two instrumentation levels.

Some studies have reported that gender may not play a role in VRFs on endodontically treated teeth; on the contrary, nonendodontically treated teeth VRFs seem to occur more frequently in male patients than females. This may be closely be related to the fact that males often present stronger masticatory muscles and higher bite force values.

Although vertical root fracture (VRF) is mostly found in endodontically treated teeth, it may also occur spontaneously. If VRF is recognized after endodontic treatment, it is considered to be iatrogenic and can lead to legal trouble. However, legal problems can be averted if the dentist can prove that the VRF existed before endodontic treatment. To determine whether a fracture is iatrogenic or spontaneous, gutta-percha will be found in the fracture line of the transversely sectioned root (after tooth extraction), and it appeared to have penetrated to the fracture line through the generated filling force [21].

Some studies suggest that endodontic treatment procedure, in which some tooth structure is inevitably removed, may weaken the treated tooth, simultaneously increasing the fracture risk even in younger patients [24], which was the case for our patient of only of 14 years of age.

### 3.3. Treatment and Prognosis

In a multi rooted tooth with VRF, root resection (amputation or hemisection) can save the tooth. However, in single rooted teeth with VRF, the prognosis is unfavorable.

Extraction may be required (because of extensive bone loss and uncertain prognosis).

However, many innovative attempts to treat and retain anterior teeth have been described in various case reports [1, 19, 22].

#### 3.3.1. Extraction and Replantation after Bonding

Studies have reported successfully treating tooth with VRF by atraumatically extracting the fractured tooth, bonding the fragments, and then replanting the tooth either directly or with a 180 degree rotation (especially in cases of anterior teeth). It was advocated that deep and narrow periodontal pockets along the fracture line may remain if teeth with VRF are replanted without rotation as intentional rotational replantation aims to avoid contact with the area where bone and periodontal ligament were lost in the treatment of VRF. The rotation of the tooth was suggested to connect the remnants of the healthy periodontal membrane, remaining on the root, with the connective tissue of the periodontally involved socket wall.

Other treatment options like the use of composite resins, mineral trioxide aggregate, and glass ionomer cements for bonding the fracture line have also been tried [20, 25].

The use of CO2 and Nd:YAG laser to fuse fractured tooth roots was also reported [19].

The Use of dual-cured adhesive resin cement is preferred for bonding the fractured fragments, as it is easy to apply and has a controlled polymerization [26].

In the present case, the extraction of tooth number 11 and its replacement by a removable prosthesis until the age of eighteen was discussed with the patient. However, the patient was reluctant for extraction and an alternative treatment plan was established which included bonding the fragments with a fluid resin cement without extraction and intentional replantation. The use of adhesive resin with intentional replantation has been reported in the literature for complete and incomplete vertical root fractures. Arikan et al. and Dogan et al. reported successful treatment outcome of vertically fractured incisors while adopting this method [27, 28].

The main objective of the treatment adopted was to preserve the natural tooth despite the uncertain prognosis so that bone support of the tooth is maintained (until the age of 25) along with its normal occlusion. This will genuinely be very useful and important for possible prosthetics or implant treatment needed in the future for this young patient.

However, failure was observed when the same method was used to treat vertically fractured posterior teeth. The possible reasons behind that could include lower occlusal forces applied on the anterior teeth along with a better maintenance of the gingival health in this area. This finding encouraged us to choose the treatment option of bonding and monitoring for the presented case of VRF in the anterior tooth.

Many of the treatment options reported involve extensive procedures often with poor outcomes. Where successful outcomes have been claimed, the long term prognosis has yet to be proven. All the case reports published so far that describe a treatment rationale do not include enough teeth to ascertain the efficacy of any procedure.

Therefore, there is a need for further clinical research on the treatment of teeth with VRF.

## References

[1] Dhawan A., Gupta S., Mittal R. (2014). Vertical root fractures: an update review. *Journal of Restorative Dentistry*.

[2] Liao W.-C., Tsai Y.-L., Wang C.-Y. (2017). Clinical and Radiographic characteristics of vertical root fracture in endodontically and nonendodontically treated teeth. *Journal of Endodontics*.

[3] García-Guerrero C., Parra-Junco C., Quijano-Guauque S., Molano N., Pineda G. A., Marín-Zuluaga D. J. (2017). Vertical root fractures in endodontically- treated teeth: a retrospective analysis of possible risk factors. *Journal of Investigative and Clinical Dentistry*.

[4] Takeuchi N., Yamamoto T., Tomofuji T., Murakami C. (2009). A retrospective study on the prognosis of teeth with root fracture in patients during the maintenance phase of periodontal therapy. *Dental Traumatology*.

[5] Chang E., Lam E., Shah P., Azarpazhooh A. (2016). Cone- beam computed tomography for detecting vertical root fractures in endodontically treated teeth: a systematic review. *Journal of Endodontics*.

[6] Yahyazadehfar M., Ivancik J., Majd H., An B., Zhang D., Arola D. (2014). On the mechanics of fatigue and fracture in teeth. *Applied Mechanics Reviews*.

[7] Karygianni L., Krengel M., Winter M., Stampf S., Wrbas K. T. (2014). Comparative assessment of the incidence of vertical root fractures between conven- tional versus surgical endodontic retreatment. *Clinical Oral Investigations*.

[8] Talwar S., Utneja S., Nawal R. R., Kaushik A., Srivastava D., Oberoy S. S. (2016). Role of cone- beam computed tomography in diagnosis of verti- cal root fractures: a systematic review and meta- analysis. *Journal of Endodontics*.

[9] Hassan B., Metska M. E., Ozok A. R., van der Stelt P., Wesselink P. R. (2009). Detection of vertical root fractures in endodontically treated teeth by a cone beam computed tomography scan. *Journal of Endodontics*.

[10] de Menezes R. F., de Araújo N. C., Rosa J. M. C. S. (2016). Detection of vertical root fractures in endodontically treated teeth in the absence and in the presence of metal post by cone-beam computed tomography. *BMC Oral Health*.

[11] Dua D., Dua A. (2015). Reconstruction and intentional replantation of a maxillary central incisor with a complete vertical root fracture: a rare case report with three years follow up. *Journal of Clinical and Diagnostic Research*.

[12] Martinsa J. N. R., Pedro Canta J., Coelho A., Baharestani M. (2014). Vertical root fracture diagnosis of crowned premolars with root canal treatment–Two case reports. *Revista Portuguesa de Estomatologia, Medicina Dentária e Cirurgia Maxilofacial*.

[13] Lustig J., Tamse A., Fuss Z. (2000). Pattern of bone resorption in vertically fractured, endodontically treated teeth. *Oral Surgery, Oral Medicine, Oral Pathology, Oral Radiology, and Endodontology*.

[14] Sugaya T., Kawanami M., Noguchi H., Kato H., Masaka N. (2001). Periodontal healing after bonding treatment of vertical root fracture. *Dental Traumatology*.

[15] Nogueira Leal da Silva E. J., Romão dos Santos G., Liess Krebs R., de Souza Coutinho-Filho T. (2012). Surgical alternative for treatment of vertical root fracture: a case report. *Iranian Endodontic Journal*.

[16] Kishen A., kumar G. V., Chen N. N. (2004). Stress-strain response in human dentine: rethinking fracture predilection in post core restored teeth. *Dental Traumatology*.

[17] Letchirakam V., Palamara J. E., Messer H. H. (2003). Finite element analysis and strain gauge studies of vertical root fracture. *Journal of Endodontics*.

[18] Devale M. R. (2017). Effect of instrumentation length and instrumentation systems: hand versus rotary files on apical crack formation. *Journal of Clinical and Diagnostic Research*.

[19] Arakawa S., Cobb C. M., Rapley J. W., Killoy W. J., Spencer P. (1996). Treatment of root fracture by Co_2_ and ND: YAG lasers: an in vitro study. *Journal of Endodontics*.

[20] Hasegawa A., Bando H., Fukai K., Vongsurasit T., Tsuchida T. (1988). Periodontal surgical approach to the vertical fracture of the root: the application of composite resin to the fractured root surface. *Nihon Shishubyo Gakkai Kaishi*.

[21] Lim M. J., Kim J. A., Choi Y., Hong C. U., Min K. S. (2017). Differentiating spontaneous vertical root fracture in endodontically treated tooth. *European Journal of Dentistry*.

[22] Barkhordar R. A. (1991). Treatment of vertical root fractures: a case report. *Quintessence International*.

[23] Tamse A. (1988). Iatrogenic vertical root fractures in endodontically treated teeth. *Dental Traumatology*.

[24] Chan C. P., Lin C. P., Tseng S.-C., Jeng J.-H. (1999). Vertical root fracture in endodontically versus non endodontically treated teeth: a survey of 315 cases in chinese patients. *Oral Surgery, Oral Medicine, Oral Pathology, Oral Radiology, and Endodontology*.

[25] Schwartz R. S., Mauger M., Clement D. J., Walker W. A. (1999). Mineral trioxide aggregate: a new material for endodontics. *Journal of the American Dental Association*.

[26] Oztürk M., Unal G. C. (2008). A successful treatment of vertical root fracture: a case report and 4 year follow-up. *Dental Traumatology*.

[27] Arikan F., Franko M., Gurkan A. (2008). Replantation of a vertically fractured maxillary central incisor after repair with adhesive resin. *International Endodontic Journal*.

[28] Dogan M. C., Akgun E. O., Yoldas H. O. (2013). Adhesive tooth fragment reattachment with intentional replantation: 36-month follow-up. *Dental Traumatology*.

